# The Quadruple Helix-Based Innovation Model of Reference Sites for Active and Healthy Ageing in Europe: The Ageing@Coimbra Case Study

**DOI:** 10.3389/fmed.2018.00132

**Published:** 2018-05-08

**Authors:** João O. Malva, Alda Amado, Alexandra Rodrigues, Anabela Mota-Pinto, Ana F. Cardoso, Ana M. Teixeira, Ana Todo-Bom, António Devesa, António F. Ambrósio, António L. Cunha, Bárbara Gomes, Carina Dantas, Cidalina Abreu, Isabel Santana, Jean Bousquet, João Apóstolo, Lúcia Santos, Lúcio Meneses de Almeida, Maddalena Illario, Rafaela Veríssimo, Vitor Rodrigues, Manuel T. Veríssimo

**Affiliations:** ^1^Ageing@Coimbra, EIP on AHA Reference Site, Coimbra, Portugal; ^2^Faculty of Medicine, Coimbra Institute for Clinical and Biomedical Research (iCBR), and CNC.IBILI, University of Coimbra, Coimbra, Portugal; ^3^Instituto de Segurança Social, Centro Distrital de Coimbra, Coimbra, Portugal; ^4^Comissão de Coordenação e Desenvolvimento Regional do Centro, Coimbra, Portugal; ^5^Centre of Investigation in Environment, Genetics and Oncobiology (CIMAGO), Faculty of Medicine, University of Coimbra, Coimbra, Portugal; ^6^Faculty of Medicine, General Pathology Institute, University of Coimbra, Coimbra, Portugal; ^7^Centre for Evidence Based Practice, Health Sciences Research Unit: Nursing (UICISA: E), Nursing School of Coimbra, Coimbra, Portugal; ^8^Research Center for Sport and Physical Activity, Faculty of Sport Sciences and Physical Education, University of Coimbra, Coimbra, Portugal; ^9^University of Coimbra Hospital, CHUC, Coimbra, Portugal; ^10^Municipality of Coimbra, Coimbra, Portugal; ^11^Pedro Nunes Institute, Laboratory of Automatics and Systems, Coimbra, Portugal; ^12^King’s College London, Cicely Saunders Institute of Palliative Care, Policy and Rehabilitation, London, United Kingdom; ^13^Caritas Coimbra, Coimbra, Portugal; ^14^Center for Neuroscience and Cell Biology (CNC), University of Coimbra, Coimbra, Portugal; ^15^Contre les Maladies Chroniques pour un VIeilissement Active (MACVIA) en France en EIP on AHA Reference Site, Montpellier, France; ^16^Portuguese Pharmaceutical Society – Center Region Branch, Coimbra, Portugal; ^17^Regional Health Authority, ARS Centro, Coimbra, Portugal; ^18^Division for Health Innovation, Campania Region and Federico II University Hospital Naples (R&D and DISMET), Naples, Italy; ^19^Centro Hospitalar de Gaia/Espinho, Gaia, Portugal

**Keywords:** ageing (aging), European commission, innovation, active and healthy aging, reference sites

## Abstract

Challenges posed by demographic changes and population aging are key priorities for the Horizon 2020 Program of the European Commission. Aligned with the vision of the European Innovation Partnership on Active and Healthy Ageing (EIP on AHA), the development, exchange, and large-scale adoption of innovative good practices is a key element of the responses required to ensure all European citizens remain as active and healthy as possible as they age. Urged by the need of developing scalable disruptive innovation across Europe, the European Commission and the EIP on AHA created the Reference Sites; local coalition of partners that develop good practices to support AHA. Ageing@Coimbra is an example of how this can be achieved at a regional level. The consortium comprises over 70 institutions that develop innovative practices to support AHA in Portugal. Ageing@Coimbra partners support a regional network of stakeholders that build a holistic ecosystem in health and social care, taking into consideration the specificities of the territories, living environments and cultural resources (2,243,934 inhabitants, 530,423 aged 65 or plus live in the Centre Region of Portugal). Good practices in reducing the burden of brain diseases that affect cognition and memory impairment in older people and tackling social isolation in urban and rural areas are among the top priorities of Ageing@Coimbra. Profiting from the collaborative work of academia, business companies, civil society, and authorities, the quadruple helix of Ageing@Coimbra supports: early diagnosis of frailty and disease; care and cure; and active, assisted, and independent living. This paper describes, as a Community Case Study, the creation of a Reference Site of the EIP on AHA, Ageing@Coimbra, and its impact in Portugal. This Reference Site can motivate other regions to develop innovative formulas to federate stakeholders and networks, building consortia at regional level. This growing movement, across Europe, is inspired by the quadruple helix concept and by the replication of innovative good practices; creating new Reference Sites for the benefit of Citizens.

## Introduction

Europe faces the global challenge of population aging with strong impact in the quality of life of individuals and families, society cohesion, health, and economic resources of citizens and member states. The percentage of people aged 65 or over in the population is expected to grow from 18.4% in 2013 to 28.4% in 2060 for the average of European countries (1). The growth will be particularly evident in the oldest old (aged 80 or over), who will increase from 5.1% in 2013 to 11.8% in 2060. European projections of old-age dependency ratios (people aged 65 or over versus people aged 15–64) reveal that the current ratio of 1 person aged 65 or over per 4 working-age people will rise to a ratio of 1 person aged 65 or over to 2 working-age people in 2060 (1). This poses huge challenges for sustaining retirement and care in later life.

Portugal stands in a particular challenging position in the overall EU28 scenario. The Portuguese population is aging rapidly with an Ageing Index (people aged 65 or over per 100 people aged less than 15) of 143.9 in 2015 compared to 27 in 1960.^1^ The percentage of people aged 80 years or over was 5.4% in 2013 and it is expected to grow to 16.1% in 2060, reaching the highest value of all EU28 countries (1).

Within Portugal, the population living in the Centre Region is particularly old due to the increase in life expectancy, migration of young adults and decrease in birth rate. Within the Centre Region, some areas are already facing the demographic scenarios expected for the European landscape in 2060, e.g., municipalities with Ageing Index above 700.^2^

Taking together, the demographic landscape of Europe, with a particularly and striking case in Portugal, urges the need of defining new strategies and implementing actions to deliver innovative and disruptive solutions. These solutions are expected to have impact at the individual, family, labor, societal, and economic dimensions to support individual health and well-being, at all ages, and deliver solutions to support health and social care sustainability. As we will elaborate bellow, the EIP on AHA movement, and the particular case of Ageing@Coimbra (as a Community Case Study), may be inspiring for the required change management processes, delivering solutions for the challenges of aging and opening new opportunities for economic growth.

## Background and Rationale

### The European Innovation Partnership on Active and Healthy Ageing

The EIP on AHA^3^ is a coalition of European partners promoted by the European Commission to develop, implement, and replicate at large-scale innovative good practices to support AHA of European citizens.

The EIP on AHA grew from recognition that the quality of life of older people is strongly affected by life style habits across the life cycle in all ages. Many older people suffer from multiple chronic conditions that demand continuous support by medical and social care providers and limit functional independence. Ultimately, poor health in later life strongly impacts on health and economic resources, human suffering, human dignity of individuals, and families (2).

The EIP on AHA fosters collaborative work of European partners to support three main strategic pillars of AHA: (1) prevention, screening, and early diagnosis; (2) care and cure; (3) independent living and active aging. EIP on AHA partners are organized in six action groups: A1, adherence to prescription and medical plans; A2, personalized health management and prevention of falls; A3, lifespan health promotion and prevention of age-related frailty and disease; B3, replicating and tutoring integrated care for chronic diseases; C2, development of interoperable independent living solutions; D4, innovation for age-friendly buildings, cities and environments.

The main goal is to achieve a triple win for Europe: (1) Health and quality of life of European citizens; (2) Sustainable and efficient care systems; and (3) Growth and expansion of EU industry.

EIP on AHA is a wide interdisciplinary and transdisciplinary network that seeks innovative collaborative models, removing silos and developing citizen-centered processes. EIP on AHA partners joint academia, health, and social care providers, citizens and patient associations and several other stakeholder groups with innovators, entrepreneurs, and business partners. The aim is to deliver knowledge and user-based competence into innovative good practices, products, and services. Only disruptive innovation and knowledge-based added value will deliver efficient meaningful large-scale interventions to face the societal challenges, opening, at the same time, new opportunities for entrepreneurs, business activity, and job creation (3, 4).

### The Reference Sites Collaborative Network

Reference Sites have been defined by the EIP on AHA as “inspirational ecosystems, delivering creative and workable solutions that improve the lives and health of older people.”^4^ Reference Sites effort is to foster and create innovative solutions and scale-up and replicate these solutions across Europe.

Reference Sites include regions, cities, integrated hospitals, or care organizations that focus on innovative and holistic approaches to support AHA. Reference Sites show diverse levels of impact and maturity toward the goal standard of regional ecosystems aligning actors across the quadruple helix: academia, authorities, business, and civil society. The impact of the activities of Reference Sites provides evidence of innovative components, and indicators are measurable in line with the Monitoring and Assessment Framework of the EIP on AHA (MAFEIP), under the dimensions “Quality of life,” “Sustainability of health-care systems,” and “Economic growth and jobs.”^5^

The first call for Reference Sites by the European Commission resulted in the award of 32 Reference Sites in 2013. The second call led to a total of 74 Reference Sites in 2016. Recently (November, 2017), the Reference Sites launched a non-profit association based in Brussels to promote synergies and collaborative work (e.g., twinning activities)—the Reference Site Collaborative Network (5).

## Methods

### The Ageing@Coimbra—Creation of a Bottom-Up Innovative Quadruple Helix-Based Consortium

The Ageing@Coimbra Consortium^6^ was formally created in January 2013 as a response of the Centre Region of Portugal to the first call for Reference Sites to develop and implement innovative good practices to support AHA.

Ageing@Coimbra is an informal network of 5 founder and 65 associated partners that build a “quadruple helix”, aligning: (1) public institutions (e.g., municipalities, health and social care authorities), (2) academia (e.g., universities and research institutions), (3) industry partners (e.g., business companies, business incubators, and clusters), and (4) civil society organizations (e.g., third age universities, cultural, and civic organizations). The consortium was founded under a leadership initiative of the University of Coimbra, the Municipality of Coimbra, the Regional Health Authority, the University of Coimbra Hospital, and the Pedro Nunes Institute (technology transfer, business incubator and business accelerator affiliated with the University of Coimbra). The Administrative Authority of the Centre Region of Portugal (“Comissão de Coordenação e Desenvolvimento Regional do Centro,” CCDRC) has been a major supporter and driver for the success of Ageing@Coimbra, actively engaging in the implementation of activities, international visibility and ownership of responsibility to include AHA in the key priorities of the Smart Specialization Strategy (RIS3) of the Center Region of Portugal. In 2017, CCDRC launched a regional competition to award three cases of good practices in three clusters: Knowledge, Health, and Life.

Ageing@Coimbra was the first EIP on AHA Reference Site in Portugal. The innovative formula and lean governance structure created an inclusive partnership with agility, rapid implementation, and growth potential. Concordantly, other regions in Portugal (e.g., Porto metropolitan area, Algarve) and regions in other European countries (e.g., Asturias in Spain, Lodz in Poland) embraced Ageing@Coimbra example to replicate the model and launched new reference sites in 2016, or they will do so in a future call expected for 2019. The same example has replication potential in other countries in Europe and globally (e.g., Northeast Brazil).

## Results

### The Ageing@Coimbra, Consortium—Reference Site in Portugal

As an active member of EIP on AHA Action Groups, Ageing@Coimbra participates in the collaborative activities and delivers success cases of projects and good practices.

#### A1 Action Group: Adherence to Medical Plans

Multimorbidity and the related prescription of multiple drugs are becoming common problems among older people, frequently associated with poor health outcomes, being inadequate and/or poor adherence to medical plans one of the relevant factors that compromise the success of treatments (6). Therefore, in agreement with the overall goals and triple win defined by the EIP on AHA, the A1 Action Group aims to contribute to the improvement of adherence to medical plans and medication at European level. Ageing@Coimbra has been actively involved in this Action Group and, since 2015, is coordinating the work and activities of one of the four strategic objectives agreed for this group’s renovated Action Plan, related to the reinforcement of the multidisciplinary and integrated approach to improve adherence to prescription and to tackle inappropriate polypharmacy.

**Figure 1:**
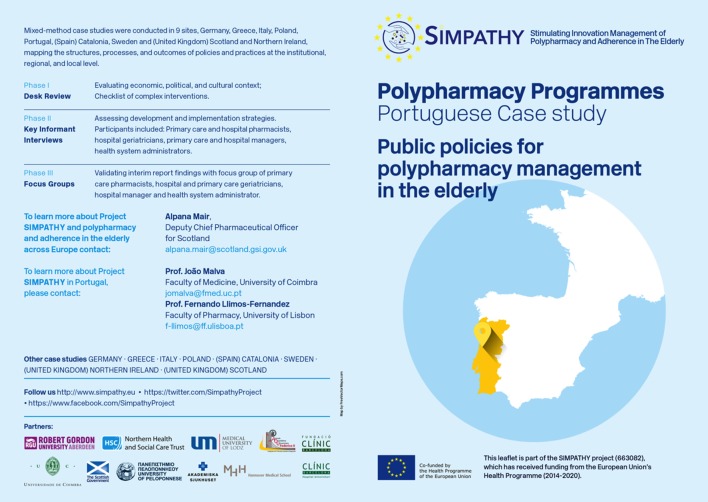
Flyer of SIMPATHY case study in Portugal. SIMPATHY in Portugal recognized the lack of policy guidelines for polypharmacy management across the country.

The collaborative work of Ageing@Coimbra in the A1 Action Group resulted in the successful development of the third Health Program Project SIMPATHY (Stimulating Innovative Management of Polypharmacy and Adherence in the Elderly), a project coordinated by the Scottish Government (Grant Agreement 663082)^7^ (6–8) (Figure 1).

#### A2 Action Group: Personalized Health Management and Prevention of Falls

The relevance of the topic addressed in the A2 Action Group is supported by the Joint Declaration of the European Stakeholders Alliance for Active Ageing through Falls Prevention^8^ “*falls are a major indicator of increasing frailty and loss of independence and mobility in older people*.” The main objective of the A2 Action Group has been to deliver EU wide evidence-based, validated and operational programs for the prevention, early identification, and minimization of risk and management of falls in order to reduce falls and the personal, system, and societal consequences of fall-related injuries. In order to reach relevant joint achievements and goals four different areas of collaborative work are being developed: (1) implementation of an integrated and person-centered service pathway for fall prevention and management; (2) data collection for evidence-based interventions on falls; (3) awareness, information, and education, to support the training of the workforce; (4) Governance: innovation, sustainability, and scaling-up, including models for exploitation, business analysis, and strategies for falls prevention technologies take up.

The collaborative work of Ageing@Coimbra in the A2 group resulted in joint activities and collaborative work in the Prevention of Falls Network for Dissemination (ProFouND), an EC funded initiative dedicated to the dissemination and implementation of best practices in falls prevention across Europe.^9^

Also, within the scope of the A2 action group, the Project “Good practices to develop physical activity programs at work—FitWork” was developed within the framework of the ERASMUS + SPORT program aimed at developing good practices to implement physical activity programs at work attending to specific risks of workplaces (muscular–skeletal disorders and sedentary workplaces). The project is coordinated by Instituto de Biomecánica de Valencia and more information can be found at.^10^

#### A3 Action Group: Lifespan Health Promotion and Prevention of Age-Related Frailty and Disease

Reflecting a growing awareness of the need for better care and sustainable and effective health services, the A3 Action Group aims to contribute to AHA with a life-course approach, implementing the shift from reactive disease management to anticipatory care of functional decline through community-based prevention, early diagnosis, and screening programs, and integrated care management systems. This patient-centered approach involving social and health-care systems is based on innovative solutions that support interactions in the areas of nutrition, frailty and functional decline, cognitive decline and physical activity. It also fosters the development of networks strengthening education and training of the health workforce, citizens empowerment through health and ICT literacy, and advancing knowledge on the determinants of frailty and chronic diseases to support early detection and sustainable interventions. The joint effort of A3 partners is the improvement and alignment of services delivered to the needs of older populations and enabling caregivers’ education and empowerment.

To ensure the scalability of the EIP on AHA commitments on frailty, the FOCUS project (*Frailty Management Optimization through EIPAHA Commitments and Utilization of Stakeholders’ Input*) was launched (9). This project aims at a critical reduction of frailty burden in Europe by deploying knowledge and tools on frailty diagnosis/screening and management, and by supporting good practices developed in this context. The FOCUS Consortium consists of a multidisciplinary team representing Centre-North (the Netherlands) and South (Spain and Italy), East (Poland), and West (UK, Portugal) Europe, under the coordination of the University of Valencia (Grant Agreement 664367). More information can be found at http://focus-aha.eu/home.

#### B3 Action Group: Integrated Care

The B3 Action Group main goal is to provide integrated care to older people with chronic conditions through a people-centered approach, foreseeing improvements in quality of life by implementing integrated care programs to reduce unnecessary hospitalization and improve the efficiency of health care. These programs are multidisciplinary, well-coordinated, accessible, anchored in the community and home care settings, and stimulate the interplay between health and social systems, industry, academia, and health-care users in order to establish innovative responses to the challenges imposed by aging.

**Figure 2:**
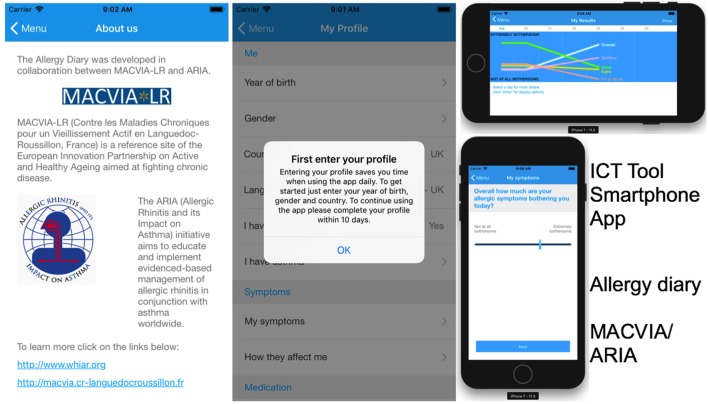
MACVIA/ARIA allergy diary Smartphone Application (App) developed by the MACVIA-Fr Reference Site, adopted and replicated across EU and non-EU countries; 15931 users—AT 655; AU 196; BE 164; BR 2039; CA 34; CH 905; CZ 5; DE 814; DK 151; ES 736; FI 456; FR 1029; GB 962; GR 240; IT 1970; LT 394; MX 905; NL 603; PL 968; PT 2264; SE 174; TR 260 (November 22, 2017).

Within the scope of the B3 Action Group, Ageing@Coimbra and Porto4Health References Sites collaborate as the key Portuguese partners of MACVIA-Fr Reference Site in a Twinning project on the large-scale deployment of a Smartphone Application (App) to monitor and control the symptoms of allergic rhinitis (5, 10) (Figure 2).

#### C2 Action Group: Independent Living Solutions

The objective of the C2 Action Group is to develop interoperable independent living solutions, including guidelines for business models. This should boost the deployment of open and personalized solutions for active and independent living that are supported by global standards and new evidence on the return of investment. The C2 Action Group provides essential input to the creation of a new market for cost-effective products and services that help older people live a more active and independent life. This reinforces ongoing research and innovation activities in Europe (and elsewhere), supported through public–private partnerships.

Within the scope of the C2 Action Group, in 2017, the AAL Forum, the biggest European event in the field of Technologies for AHA was organized in Coimbra, by the Pedro Nunes Institute, in partnership with the Portuguese Foundation for Science and Technology and some of the Ageing@Coimbra core partners. The theme of the Forum was “Bridging the gaps between technology solutions and aging well. What can YOU do?” Interoperable solutions were discussed as crucial to assure the value of the digital solutions, for example contributing for the integration between social and health care.

**Figure 3:**
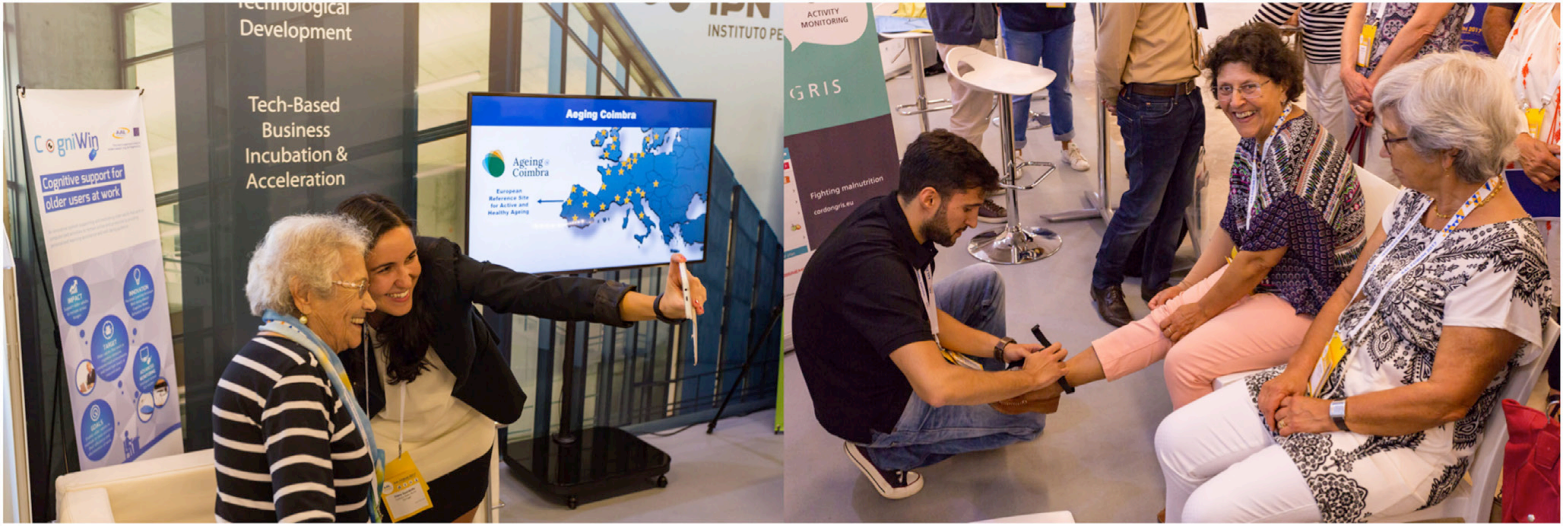
Older citizens (identified through Third Age Universities and Social Care Providers) experience and evaluate technologies at the AAL Forum 2017 (Coimbra, October 2–4, 2017). Over 120 old citizens tested, evaluated, and provided recommendations for technology developers. The identified participants and the AAL Forum organization expressed written agreement for the inclusion of these photos in the present publication.

An inspirational fresh addition to this year’s AAL Forum exhibition hall was the attendance of over 120 end-users above the age of 65, all eager to give their honest opinion on what they saw. Exhibitors had approximately 7 min to present and sell their AAL solution to the guests, which turned out to be more of a challenge than the majority of exhibitors expected (Figure 3). After visiting each of the exhibition stands, the group of over 65’s had the hard job of picking their three favorite stands.

For the first time in the AAL Forum, the jury responsible for awarding the Exhibition Prize was constituted only by end-users above the age of 65. This activity was supported by Ageing@Coimbra and the Administrative Authority of the Centre Region of Portugal.

Another example of the collaborative work of Ageing@Coimbra members within the scope of C2 Action Group is well represented by the project GrowMeUp, funded under the H2020 call PHC-19-2014 “Advancing active and healthy aging with ICT: service robotics within assisted living environments”; coordinated by the University of Coimbra and including the active partnership with Caritas Coimbra as an end-user organization (Grant Agreement 643647).^11^

#### D4 Action Group: Age-Friendly Environments

The D4 Action Group has its main roots on the concept of Age-Friendly Environments, as envisaged by the World Health Organization^12^ (WHO), referring to physical and social environments as key determinants for health, well-being and the participation of people as they age. In that sense, the main objective of the D4 Action Group is to contribute to a more inclusive society and communities across Europe by empowering older persons through scaled up inclusive solutions.^13^ Caritas Coimbra is main coordinator of the domain 8 of this action group—connect and leverage local and regional age-friendly environments—which fosters wide participation of different stakeholders across Europe, supporting networking, transfer of experience and the upscale of good practices.

Caritas Coimbra is also a founder member and Vice-President of the European Covenant on Demographic Change, an organization that aims to gather local, regional, and national authorities as well as other stakeholders in order to cooperate and implement evidence-based solutions to support AHA.^14^ The Covenant comprises more than 150 members—local and regional authorities, industries, research centers/universities, civil society organizations—to link up, benefit from each other’s experience and work together to promote initiatives on age-friendly environments across the EU.

The collaborative work of Ageing@Coimbra in D4 group includes the leadership of the University of Coimbra in the H2020 project Euro-Healthy (Shaping European Policies to Promote Health Equity) with a key goal to deliver a Population Health Index and a Web Geographic Information System (Web-GIS) addressing health inequity across the EU (Grant Agreement 643398) (11).^15^

## Ageing@Coimbra Innovation Clusters on Brain Ageing and on Social Isolation

### Ageing@Coimbra Provides an Integrated Pipeline in Biomarkers for Neurodegenerative Diseases

The area of biomarkers for neurodegenerative diseases, particularly concerning Alzheimer’s disease, profits from the solid basis of neuroscience research provided by CNC.IBILI. Fundamental research is transferred from bench to bedside by means of knowledge-based genetics, biochemical, imaging, and neuropsychology biomarkers of cognitive dysfunction.

Genetics and biochemical biomarkers currently used to identify risk factors and biomarkers of Alzheimer’s disease include APP, tau, amyloid beta 1-42, genotyping for PSEN1, PSEN2, APP, ApoE (12).

The Faculty of Psychology of the University of Coimbra provides neuropsychology tests to screen for functional and memory competence of older adults including Adults and Older Adults Functional Assessment Inventory (IAIFA), World Health Organization Quality of Life-OLD module, Geriatric Depression Scale (GDS-15), cognitive performance (MOCA, ACE-R).

The early diagnosis pipeline is complemented with robust brain imaging tools to ensure high quality diagnosis of putative neurodegenerative conditions. The Applied Nuclear Sciences Institute (ICNAS) pipeline launches innovative cyclotron-based synthesis methods of radiotracers including PET/FDG, PET/PiB, PET/PK11195, and other radiotracers upgrading the diagnosis tools.

This integrated approach, aligning technologies and professionals/resources for a better public service results in relevant assets for Ageing@Coimbra stakeholders, creating the most valuable and reliable integrated health service in Alzheimer’s diseases in Portugal; with recognized impact in the Health Cluster Portugal, the Joint Program in Neurodegenerative Diseases, and the EIT Health Knowledge Innovation Community (KIC).

The diagnostics pipeline for early detection of Alzheimer’s disease contributes to better health-care management and also to liaise with patient associations (e.g., Alzheimer’s disease; Alzheimer Portugal) for better social care and support to patients and care providers.

### Social Isolation—Mobile Units, Continuous Integrated Care, and Healthy Food

The territory of the Center Region of Portugal offers peculiar conditions as a regional living laboratory. In the region, about 23% of residents are aged 65 or over living in diverse environments, urban versus rural, highly dense versus low-density territories. In inland areas, especially in the mountain area, low-density territories also create geographic barriers to mobility especially of frail people contributing to social isolation. On the other hand, social isolation is also a burden of older persons living alone in urban areas of the Centre Region of Portugal.

These particular conditions create a local need to develop integrated care and personalized solutions to support older people living in socially challenging conditions.

The majority of municipalities in the Region have been equipped with health mobile units to deliver primary care and social care support to frail older people living in rural isolated areas. The added value of these services is well recognized by users and professionals, showing increase demand in terms of individual requests by the citizens and attracted interest by other municipalities.

Portugal launched in 2016 a new coordination structure of the National Network for Integrated Care Units. This network provided complementary health and social care support to populations, being socially isolated frail older people the main beneficiaries. The regional network includes 3 main units for palliative care, 9 units for convalescence care, and more than 62 integrated home care teams. Ageing@Coimbra links with integrated home care teams in close collaboration with the regional health authority, the national insurance office, and municipalities.

The social support to isolated frail older people in the region is a key priority for local players, including municipalities. Several important actions support intergeneration activities to tackle social isolation, ICT literacy, polypharmacy, and adherence to therapy strategies, assisted living programs, innovative strategies to engage older citizens with meaningful activities, and an humanitarian approach in health care—both in rural and urban areas.

The regional health authority launched a program (“pão.come”) to reduce the content of salt in the daily food of Portuguese citizens, in particular the amount of salt in bakery. Traditionally, Portuguese citizens use excess salt in food, specially in bakery. This fact has been associated with high-prevalence of cerebrovascular diseases (13). Interestingly, the population of the Centre Region faces a major change in the profile of cerebrovascular diseases. The number of fatalities and permanent impairment conditions has been reduced significantly. Two major driving forces can account for the success of the “pão.come” program: (1) effective reduction of salt in bread that contributes to reduce the global numbers of stroke and cerebrovascular conditions; (2) the implementation of special priority channels (green way) in hospitals for people suffering from stroke and acute cerebrovascular diseases.

**Figure 4:**
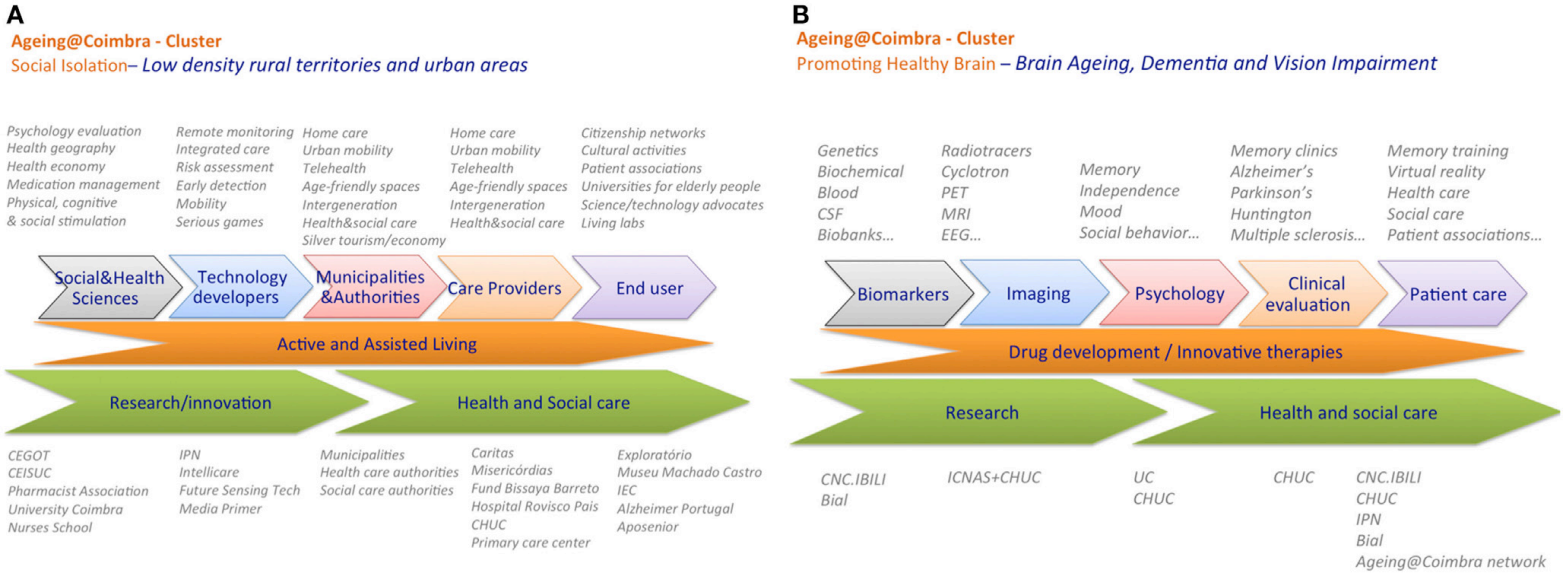
Innovation cluster of Ageing@Coimbra on **(A)** “Social isolation: Low-density territories and urban areas,” **(B)** “Promoting Healthy Brain: Brain aging, dementia, and vision impairment.” The innovation pipeline represents the integration of stakeholder’s activities from fundamental research into clinical or end-user applications.

A schematic representation of the innovation clusters of Ageing@Coimbra (promoting healthy brain; social isolation) is depicted in Figure 4.

## Fundamental and Applied Research in Ageing, Care and Innovation in Geriatric Medicine

### Fundamental and Applied Research

Research on the mechanisms of aging-related diseases is a solid and established research area at the University of Coimbra and at the Center for Neuroscience and Cell Biology of Coimbra. Research in this area delivers high-impact and highly cited publications, international collaborations, advanced training of Ph.D., and postdoctoral students as well as entrepreneurship training and new spin off companies. Highlighting the priority of aging research, in 2016, a new ERA Chair group (H2020 Widespread, Grant Agreement 669088) was appointed by the Faculty of Medicine of the University of Coimbra to develop a research program, advanced teaching and translational research in cardiovascular aging (14).^16^

### EIT Health

In 2015, three founder members of Ageing@Coimbra, the University of Coimbra, University of Coimbra Hospital and Pedro Nunes Institute, were accepted, as Associated Partners, in the new KIC in Active Living and Healthy Ageing from EIT Health. Together with the pharmaceutical company BIAL, they formed a cluster of Innostars, a legal entity member of EIT Health.

EIT Health is a powerful consortium of more than 140 members from academia and industry partners that develop collaborative market-driven projects to tackle societal challenges: (1) promote healthy living; (2) support active aging; (3) improve health care. Projects deliver better training of students, executives, and citizens (Campus); transform innovative ideas into products and services (Innovation Projects); and create and catapult business creation (Accelerator).^17^

### Geriatric Medicine

The University of Coimbra Hospital offers a well-established multidisciplinary geriatric assessment service. The knowledge gathered by the clinical team supported the creation of a new clinical service entirely dedicated to AHA, including daily clinical care, a hospital residence, outpatient consultations and community health-care services. The key pillars of the new unit enclose clinical care of geriatric patients, training of new health-care professionals and research on aging and geriatrics.

The clinical unit on AHA establishes strong links with other hospital specialty services for prevalent and multimorbid conditions, including Neurodegenerative and Psychiatric Diseases, Pneumology and Respiratory Diseases, Immunology, Cardiovascular Diseases, Diabetes, Cancer, Musculoskeletal Diseases, among others.

Geriatric Medicine profits from the institutional collaboration of the University of Coimbra Hospital and the Rehabilitation Hospital Rovisco Pais and other local Hospital units in the region. Dedicated patient-centered rehabilitation services profiting from holistic approaches and multidisciplinary care are key success factors to prevent and treat geriatric frailty, particularly ortogeriatric frailty. Long-term care and palliative care multidisciplinary services in the region offer support to patients and families affected by long-term functional dependence and end-of life care for older patients and their families, including a hospital palliative care team offering support across clinical services at the University of Coimbra Hospital and two inpatient palliative care units, at the Portuguese Cancer Institute—Coimbra and Cantanhede Hospital (the latter also offering home care support). Reinforcing this strategic area, the Calouste Gulbenkian Foundation supports a “Professorship in Palliative Care” at the Faculty of Medicine of the University of Coimbra to strengthen research and education in Palliative Care in the Centre Region.

### Health Literacy and Professional Training

The training of formal caregivers (working in institutions for older people), or informal caregivers (providing non-remunerated care for older people) is extremely important, especially when taking into account the growing older population that depends on care for chronic and degenerative diseases.

The University of Coimbra, together with Ageing@Coimbra, knowing the demand for specialized caregivers’ training and the need to reach an audience with time limitations and geographically dispersed, concluded that a distance learning modality (e-learning course) was among the most appropriate responses. The Distance Learning Course, “Active and Healthy Ageing,” which is a method of active learning providing training in the basic care areas of older people, was created by a team of lecturers of the Faculty of Medicine.^18^

Health literacy in aging is also a priority topic in postgraduate training. The Faculty of Medicine offers a masters degree in geriatrics and gerontology, where medical doctors can learn and upgrade their knowledge in age and age-related chronic diseases. Geriatrics in Coimbra is supported by advanced teaching, clinical health care and professional training. Advanced teaching at the Faculty of Medicine of the University of Coimbra includes the Ph.D. program in health sciences, the Ph.D. program in aging and chronic diseases, the masters in geriatrics and undergraduate training in geriatrics under the scope of the masters course in medicine. Postgraduate training of health-care professionals also includes the annual course on Ageing and Geriatrics (in 2017, we celebrate its 16th Edition).

Nursing School of Coimbra promotes the implementation of the comprehensive geriatric assessment at a national level. The activities include cultural adaptation and validation of tools for use in the field of geriatrics and participation in the development of national and international web platforms to support National and European strategy to intervene on older adults’ frailty and to involve stakeholders in this process. The measurable impact of work of Nursing School of Coimbra is also observable in prevention of cognitive decline in advanced age by promoting the implementation of cognitive stimulation-based practice in different health-care contexts and by enabling multidisciplinary teams to implement autonomously this practice in different regions of Portugal. In addition, Nursing School of Coimbra is also training health professionals at a national level by enabling them to achieve critical reasoning, questioning of the ongoing practices and seeking and evaluating information to support Evidence-Based Practice. Nursing School of Coimbra is also offering postgraduate programs in Geriatric Health and Ageing, Health, and Citizenship.

### Social Participation

Ageing@Coimbra profits from the engagement of several institutional partners that promote the social inclusion and participation of seniors in meaningful social activities and long-life learning. These institutions include Third Age Universities, Citizenship Associations, Science Centers, Museums, among others. Together, these institutions mobilize thousands of older citizens and their families in a number of activities involving the society: including heart and cardiovascular diseases awareness; Diabetes; Brain Awareness Week; International Day of the Older Person; European Scientists Night; among others.

## Good Practices Ageing@Coimbra, 2017

In 2017, the Regional Authority of Centre Region, in a partnership with Ageing@Coimbra, launched a call to award good practices supporting AHA and age-friendly territories in the 100 Municipalities of the Region. This initiative contributes to (1) identify innovative good practices; (2) publicly recognize individual and institutional stakeholders; (3) disseminate knowledge to support adoption and scaling-up in other territories. The call awarded, in 2017, three categories: (1) Life+ (“Vida+”), to support healthy life styles; (2) Health+ (“Saúde+”), supporting innovative health care for older people; and (3) knowledge+ (Conhecimento+), to support research and technology applied to AHA.

**Figure 5:**
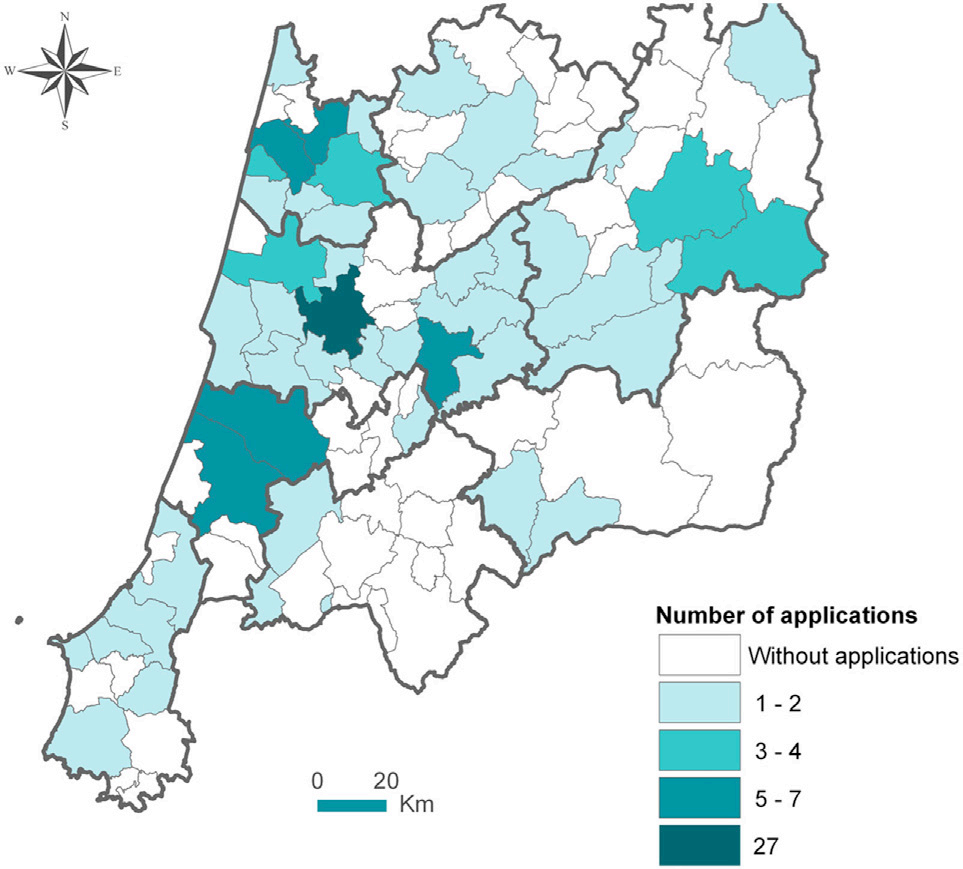
Number of applications for the award of good practices on AHA (“Boas Práticas de Envelhecimento Ativo e Saudável da Região Centro”). The contest involved stakeholders from 49 of the 100 municipalities of Centro Region, that submitted 1 or more application, per municipality, of the total of 128 good practices (blue color scale for number of application per municipality).

We received 128 valid applications from diverse thematic and geographic origins, including 74 applications for the category “Life,” 38 for “Health,” and 16 applications for “Knowledge,” from a total of 118 institutions. These institutions included 34 municipalities, 12 social care providers, and several citizenship associations and universities, from a total of 49 municipalities (Figure 5).

“Life” projects include programs for physical activity, with or without assessment of health parameters, multidimensional evaluation (clinics, social and gerontology), intergenerational dialog, therapy by arts, creation, and development of informatics platforms. The winner “Novas Primaveras,” submitted by the Arts and Music Association of Pousos village, describes a program focused on therapy for older people through arts. This good practice was launched in 2004 and involves (currently) 12,711 direct and 84 indirect users from 30 Institutions, engaging users in music, dancing, theater, poetry, and other artistic expressions.

“Health” projects enclosed areas related with new mobile health units to support home care, implementation of new methodologies of health care (primary, non-formal, long-term, dementia), prevention of frailty in older people, screening and diagnosis of disease, physical and cognitive stimulation and rehabilitation, monitoring of chronic diseases using ICT’s, support of patients in hospital and care residence settings (arts, comedy), health literacy, and health promotion. The winner good practice described specialized care for dementia patients (“Cuidados especializados para demência”) submitted by the network of care providers “União das Misericórdias Portuguesas—Unidade de Cuidados Continuados Bento XVI (Fátima)”. This dementia care residence is a pilot unit established in 2013, including environmental, professional, therapeutics competences, recognized as a reference model for the care of patients with cognitive impairment and dementia. It includes 20 beds for mid-term care and rehabilitation (up to 90 days) and 30 beds for long-term care and maintenance (up to 180 days).

“Knowledge” projects include: research and application of programs and new models; application of ICT solutions; collaborative models using telehealth and teleassistance; intergenerational approaches; infrastructures; equipment and access to knowledge; multimorbidity research and care; AHA and longevity, databases and population profiling. The winner was a project submitted by the Faculty of Medicine of the University of Coimbra and the Center for Neuroscience and Cell Biology: “NoMicro Technologies.” This project describes the development of a technology to support healing of chronic wounds and prevention of infection by pathogens. NoMicro Technologies is currently in a phase of preclinical proof of concept.

In conclusion, the number and quality of the applications received in the call exceeded the best expectations of the organizing committee, reflecting the growing involvement and awareness of stakeholders of the Centre Region to support AHA of citizens.

## Discussion

Standing on the big societal challenges created by the impact of population aging in individual’s health, but also for efficiency and sustainability of the health and social care systems, the movement created by EIP on AHA addresses the individual’s and societal hurdles, under a positive perspective of stimulating the economic growth and job creation.

The collaborative work developed by EIP on AHA partners, under the scope of the six active action groups or under the scope of the quadruple helix-based Reference Sites, has delivered an impressive number of ground-transforming good practices, innovation schemes, and change management process, with big societal impact and economic potential.^19^

EIP on AHA Reference Sites like Ageing@Coimbra have shown to be instrumental bodies for helping the European Commission in the implementation of policies, including the “Blueprint for Digital Transformation on Health and Care in an Ageing Society”^20^ and “Boosting Innovation on Active and Healthy Ageing in the Digital Single Market.”^21^ The goal of the blueprint is to reach out in 2018 more than 50 European Regions that will invest in the implementation and/or deployment of large-scale digital solutions for health and care. EIP on AHA is one of the key champions pre-identified by the European Commission that contributed to the Blueprint and committed to deliver results. The Digital Single Market Strategy unites regions, industry, and users around a shared vision on how digital innovation can transform health and care in Europe and will create the background for the impact expected for the Blueprint policy implementation. The Reference Sites are seen as key elements and stakeholders of this transformation. Accordingly, the close interaction of EIP on AHA and Reference Sites with national/regional authorities and international organizations, including WHO, foster the impact of activities leveraged from regional to European and global dimensions (5).

The impact of Reference Sites in the European landscape is also revealed by the collaborative work under the twinning collaborative scheme, involving delivery of good practices by innovator regions/actor and adoption by twin Reference Sites. The example of the twinning project lead by MACVIA and adopted/replicated by Ageing@Coimbra, and many other Reference Sites is a good example of a successful twinning project with impact in the society (see text footnote 20).

The challenges imposed to societies by population aging and demographic changes are of paramount complexity. Innovative formulas to deliver practices and services and organization models, like those embedded in EIP on AHA Reference Sites and Ageing@Coimbra, might prove to be key elements of the change management schemes needed to deliver quality of life to citizens and sustainability of health and care systems, starting from pilot twinning schemes to large scale-up replication across Europe and Globally.

## Conclusion

In conclusion, the innovative and successful example of Ageing@Coimbra as a Reference Site of EIP on AHA supports the adoption of bottom-up, inclusive, and holistic approaches to create regional networks and partnerships with the aim of supporting the creation and replication of good practices for Active and Healthy Ageing. This methodology has proven to be successful in Coimbra/Centro de Portugal and other regions of Portugal, as well as in other European countries. Other examples of innovative regions embracing this model may follow and reinforce the change management objectives pursued by the EIP on AHA and the European Commission. However, important hurdles related with poor commitment of partners, oversimplification of processes in lean management structures are also difficulties that need to be constantly monitored and overcome.

Ageing@Coimbra is a growing consortium, consolidating its unique position as an aging network in the Centre Region of Portugal, with strong collaborations with other Reference Sites and international networks. Ageing@Coimbra will support the replication of good practices emerging from its stakeholder members and will twin with other European partners to foster the adoption of innovative practices and technologies for the benefit of Centre Region and European citizens.

## Author Contributions

JM: concept design, writing the text, editing figures, and manuscript correction. AA: concept design, manuscript correction. AR: concept design, writing the text, editing figures, and manuscript correction. AM-P: writing the text and manuscript correction. AFC: concept design; writing the text; and manuscript correction. AT: concept design; writing the text; and manuscript correction. AT-B: editing figures and manuscript correction. AD: concept design and manuscript correction. AFA: manuscript correction. ALC: concept design; writing the text; and manuscript correction. CD: concept design; writing the text; editing figures; and manuscript correction. BG: writing the text and manuscript correction. CA: concept design; writing the text; and manuscript correction. IS: manuscript correction. JB: editing figures and manuscript correction. JA: concept design; writing the text; and manuscript correction. LS: concept design; writing the text; and manuscript correction. LA: concept design and manuscript correction. MI: writing the text; manuscript correction. RV: manuscript correction. VR: concept design and manuscript correction. MV: concept design; writing the text; editing figures; and manuscript correction.

## Conflict of Interest Statement

The authors declare that the research was conducted in the absence of any commercial or financial relationships that could be construed as a potential conflict of interest.

## Footnotes

^1^http://www.pordata.pt/ (Accessed: April 24, 2018).

^2^https://www.ine.pt/ (Accessed: April 24, 2018).

^3^https://ec.europa.eu/eip/ageing/home_en (Accessed: April 24, 2018).

^4^https://ec.europa.eu/eip/ageing/reference-sites_en (Accessed: April 24, 2018).

^5^http://mafeip.eu/ (Accessed: April 24, 2018).

^6^www.ageingcoimbra.pt/en/ (Accessed: April 24, 2018).

^7^www.simpathy.eu (Accessed: April 24, 2018).

^8^http://www.eunaapa.org/wp-content/uploads/2015/09/Joint-Declaration_Sept-2015.pdf (Accessed: April 24, 2018).

^9^http://profound.eu.com (Accessed: April 24, 2018).

^10^www.fitwork.eu (Accessed: April 24, 2018).

^11^www.growmeup.eu (Accessed: April 24, 2018).

^12^http://www.who.int/ageing/projects/age-friendly-environments/en/ (Accessed: April 24, 2018).

^13^https://ec.europa.eu/eip/ageing/action-groups-eip-aha/d4-age-friendly-environments_en (Accessed: April 24, 2018).

^14^www.agefriendlyeurope.org (Accessed: April 24, 2018).

^15^www.euro-healthy.eu (Accessed: April 24, 2018).

^16^http://erachair.uc-biotech.pt/team/ (Accessed: April 24, 2018).

^17^www.eithealth.eu/home (Accessed: April 24, 2018).

^18^http://www.ed.uc.pt/educ/curso?id=127 (Accessed: April 24, 2018).

^19^http://www.scale-aha.eu/fileadmin/scaleaha/documents/scaleaha_d5.4_finalstudyreport.pdf (Accessed: April 24, 2018).

^20^https://ec.europa.eu/digital-single-market/en/blueprint-digital-transformation-health-and-care-ageing-society (Accessed: April 24, 2018).

^21^https://ec.europa.eu/digital-single-market/en/node/84742 (Accessed: April 24, 2018).
